# Evaluation of the combination effect of rutin and vitamin C supplementation on the oxidative stress and inflammation in hemodialysis patients

**DOI:** 10.3389/fphar.2022.961590

**Published:** 2022-09-08

**Authors:** Samia Omar, Radwa Maher El Borolossy, Tamer Elsaid, Nagwa A. Sabri

**Affiliations:** ^1^ Department of Clinical Pharmacy, Faculty of Pharmacy, Ain Shams University, Cairo, Egypt; ^2^ Department of Nephrology, Faculty of Medicine, Ain Shams University, Cairo, Egypt

**Keywords:** rutin (PubChem CID), vitamin C, inflammation markers, oxidative stress, hemodialysis

## Abstract

**Background:** Hemodialysis (HD) patients are at risk of malnutrition, cardiovascular complications, and all-cause mortality due to oxidative stress and inflammation. Some studies have demonstrated that rutin attenuates oxidative stress and inflammation in CKD rats, but its effects in HD patients are unknown to date.

**Aim:** The aim of this study was to evaluate the effect of rutin and vitamin C versus vitamin C alone on oxidative stress and inflammation in HD patients.

**Methods**: A prospective randomized, open-label, controlled trial enrolled on hundred and five HD patients divided into three groups as follows: patients in group 1 were given a rutin/vitamin C combination (Ruta C group as the combination trade name is known as Ruta C 60 tablets), patients in group 2 were given vitamin C (1 g) (vitamin C group), and group 3 was the control group; the study period was 16 weeks. The following were assessed at baseline and at the end of the study: serum malondialdehyde (MDA), glutathione peroxidase (GPx), high-sensitivity C-reactive protein (hs-CRP), tumor necrosis factor-α (TNF-α), lipid profile levels, and erythrocyte sedimentation rate.

**Results:** It was found that vitamin C significantly increased serum GPx in group 2 (*p* = 0.001) compared to a non-significant result in both group 1 and 3; in addition, serum MDA and TNF-α values had decreased significantly in the three groups compared to their baselines; however, a non-significant difference was seen among the studied groups at the end of the study. On the other hand, MDA levels were reduced by 50% in interventional groups compared to 28% in the control group, while the Ruta C group showed an 80% reduction in the level of TNF *α* compared to the 78% reduction observed in the vitamin C group, and finally, the interventional drugs showed a significant improvement in the lipid profile.

**Conclusion:** Vitamin C supplementation alone for 16 weeks had a potential effect on the antioxidant’s GPx activity. Moreover, it was reported that both vitamin C alone or the rutin/vitamin C combination (Ruta C) showed a protective role against lipid peroxidation, evidenced by the reduced levels of MDA. Finally, rutin had a favorable synergistic effect with vitamin C in reducing TG and TNF-α levels and increasing HDL-C level.

## 1 Introduction

Chronic kidney disease (CKD) is a widespread universal problem (Levey et al., 2007). Several biological/clinical functions are affected with the progressive deterioration of the kidney’s function, such as the increase in the production of inflammatory/oxidative stress mediators (Russa et al., 2019). The level of pro-oxidants is increased in CKD because the kidney is an important source of antioxidant enzymes, including glutathione peroxidase (Kelly and Rothwell, 2020). Therefore, dialysis or transplantation as kidney replacement treatments can improve both the outcome and the prognosis of end-stage renal disease (Eckardt et al., 2018).

The oxidative stress (OS) is a global challenge in life which might be exaggerated as a result of the hemodialysis (HD) procedure, where the related factors responsible for this are as follows: first, activation of leukocytes by the dialysis membrane and dialysate which in turn induces inflammation and triggers reactive oxygen species production and accumulation of oxidative products; second, the filters of HD trap the low- or the very-low-molecular-weight antioxidants; and third, dietary restrictions imposed on HD patients which aggravate the deficiency of exogenous antioxidants which in turn decreases their antioxidant function, and OS is highly related with chronic inflammation (Liakopoulos et al., 2017).

On the other hand, dietary restrictions on vegetables and fruits in hemodialysis (HD) patients may potentiate the depletion of the antioxidant defense system resembled in decreased levels of vitamin E and vitamin C, in addition to reduced levels of selenium, and reduced function of glutathione (Canaud et al., 1999; Ross, 1999). In addition, several factors induce pro-oxidant activity in HD patients, including chronic inflammation, uremic status, hypertension, obesity, dyslipidemia, diabetes, advanced age, enhanced vascular calcification, and other dialysis-related factors (Locatelli et al., 2003).

It is worthy to mention that reactive oxygen species (ROS) can be inactivated by enzyme systems and scavenged by antioxidants, on the contrary, when ROS are produced in excessive amounts, they cannot be neutralized causing cellular structure’s alterations as DNA damage, oxidation of lipids and proteins, impairment of cellular activity, and retarded enzymatic activity (Rapa et al., 2019).

It has been demonstrated that significantly enhanced oxidative stress and damage in peripheral and mononuclear leukocytes and increased plasma levels of xanthine oxidase, oxidized glutathione (GSSG), and malondialdehyde; decreased superoxide dismutase, catalase, glutathione peroxidase, and glutathione (GSH); and altered GSSG/GSH balance were found in non-dialysis-dependent-CKD, and hemodialysis patients compared to healthy controls (Vida et al., 2021).

Regarding chronic inflammation, it is considered a comorbid factor in CKD, and especially patients on hemodialysis (HD) (Akchurin and Kaskel, 2015), evidenced by the presence of cardiovascular disease in greater than 50% of patients undergoing dialysis, which is the major cause of their death (Cozzolino et al., 2018). In addition, increased inflammation and lipid peroxidation are characteristics of HD patients (Bayés et al., 2001). Handelman et al. (2001) reported significantly higher levels of F2-isoprostanes in HD patients compared to controls with normal kidney function. Also, a strong association between F2-isoprostanes and C-reactive protein levels has been found in the HD group, suggesting a tight relationship between inflammation and oxidative stress in patients with HD.

Due to the reported deficiency of vitamin C in hemodialysis patients (HD), thus, an improvement in their health may be achieved with greater intake of this vitamin (Raimann et al., 2013), which competitively reacts with reactive oxygen species scavenging and in turn is oxidized, resulting in protection of the structure of lipid membranes, proteins, and DNA from being damaged (Berretta et al., 2020). Moreover, plasma and intracellular vitamin C, being a potent antioxidant, are readily oxidized to dehydroascorbic acid, unfortunately, plasma levels of vitamin C are low in HD, while, urinary vitamin C loss is increased due to the administration of diuretics by dialysis population, where about 200 mg of vitamin C is lost in the dialysate/week (Deicher and Hörl, 2003).

Morena et al. (2002) recorded the loss of vitamin C during a hemodialysis session, leading to a deficiency of vitamin C, which in turn was associated with a high level of malondialdhyde (MDA) and reduced activity of glutathione peroxidase. Besides, it was reported that oral supplementation of vitamin C decreased oxidative stress by decreasing MDA and lipoperoxide levels while causing an increase in the antioxidant capacity of hemodialysis and CKD stage 3 and 4 patients (Ghiadoni et al., 2004). The previous studies were diverse in terms of population, doses used, follow-up length, and the markers assessed (Liakopoulos et al., 2019), thus, targeting chronic oxidative stress and inflammation is a necessary proposal to test the influence of vitamin C and put an end to their complications in HD patients (Chaghouri et al., 2021).

Concerning rutin (vitamin P) which belongs to the flavonoid group and is present in tea leaves, apples, and many more contain rutin as active component and these days has been investigated for its nutraceutical action (Ganeshpurkar and Saluja, 2017). Rutin has both anti-oxidative and anti-inflammatory effects that make it extensively utilized in pharmacological approaches (Perk et al., 2014; Al-Dhabi et al., 2015), not only that, but it also has anticancer (Webster et al., 1996), anti-diabetic (Ahmed et al., 2010), and antimicrobial (Panasiak et al., 1989) actions. It has been confirmed that rutin attenuates schizophrenia-like behavior induced by ketamine in mice through reducing nicotinamide adenine dinucleotide phosphate oxidase-2 (Nox-2) expression, oxidative/nitrergic stresses, acetylcholinesterase activity, and preventing the decrease of glutamic-acid decarboxylase-67 (GAD_67_) expression (Oshodi et al., 2021).

Interestingly, there is growing evidence that rutin has the ability to influence the cellular metabolism by affecting the inflammatory process, where, there is preclinical evidence that rutin can attenuate the inflammation by decreasing the pro-inflammatory markers levels via its strong antioxidant activity such as TNF-α, interleukin-1 (IL-1β), interleukin (IL)-6, and cyclooxygenase-2 (Muvhulawa et al., 2022). Quercetin (a metabolite of Rutin) displays a broad spectrum of antiviral activities. A recent study suggested the administration of quercetin and vitamin C combination for the prophylaxis and early treatment of infections of the respiratory tract, particularly coronavirus patients (Colunga Biancatelli et al., 2020).

The combination of rutin and vitamin C is often used in oral dosage form for the treatment of colds and flu. In addition, the use of this formulation is recommended for vascular disorders as rutin decreases the permeability of capillaries, increases their resistance, and prevents edema (Gabor, 1972).

Up to the present, the interaction of orally supplemented rutin and vitamin C has been studied and investigated with regard to their anti-inflammatory effect and vascular sealing action (Milde et al., 2004; Al-Rejaie et al., 2012), also, it has been reported that rutin enhances the reducing power of vitamin C (Afanas’ev et al., 1989). Moreover, on undergoing an *in vitro* investigation by combining rutin with vitamin C, a synergistic effect and cytoprotective ability from UVA and UVB radiation were reported, which was interpreted to occur through the inhibition of inflammatory processes approved by deceased Nuclear Factor Kappa B (NFκB) expression (Gęgotek et al., 2019).

Despite the antioxidant and anti-inflammatory effects of each component (vitamin C and rutin) individually, up to date, there has been no prospective clinical trial performed to evaluate the antioxidant and anti-inflammatory effects of the supplementation of hemodialysis (HD) patients with the vitamin C/rutin combination. This randomized clinical trial aimed to evaluate the combined effect of rutin and vitamin C supplementation compared to control and vitamin C supplementation alone on the oxidative stress and inflammatory markers in HD patients.

## 2 Methods

### 2.1 Study design

The current study design was a prospective, interventional, open-label, randomized controlled clinical trial conducted on adult patients with end-stage renal disease on regular hemodialysis at the hemodialysis unit in Ain Shams University hospital, Cairo, Egypt, from the start of August (2021) to the end of November (2021).

### 2.2 Patient eligibility (inclusion and exclusion criteria)

All patients were assessed for eligibility criteria and were included in the study only when they had met the following inclusion criteria: age greater than 18 years old, hemodialysis in the last 3 months or longer, and hemodialysis frequency of three times per week or more. On the other hand, the exclusion criteria included treatment with antioxidant agents such as vitamin C and E during the two preceding months prior to the study, active liver disease, and pregnant patients or patients planning pregnancy.

### 2.3 Enrollment and allocation

**FIGURE 1:**
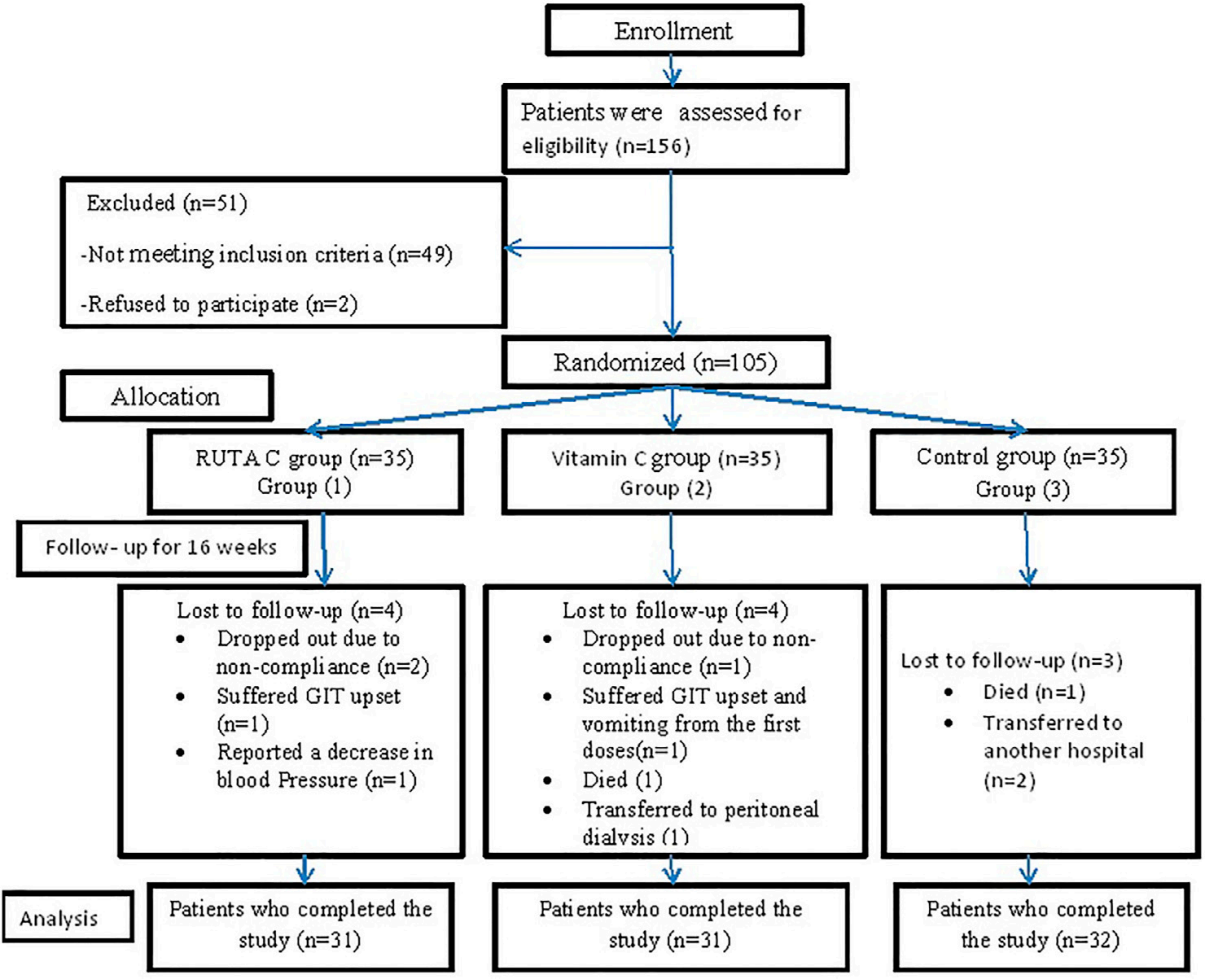
Flow diagram representing enrollment, allocation, follow-up, and analysis processes.

A total of 156 patients were assessed for eligibility; 51 patients were excluded (49 patients had not met the inclusion criteria, and two patients refused to participate in the study). One hundred and five patients were randomly allocated and distributed to the three studied groups as follows; 35 patients in the RUTA C group (group1), 35 patients to the vitamin C group (group2), and 35 patients to the control group (group3). Eleven patients all-over the three groups were withdrawn and could not complete the study due to different reasons as described in Figure 1.

### 2.4 Treatment intervention

Eligible hemodialysis patients were randomized into three groups. The first group (group1) included 35 patients who received a dose of two tablets of rutin 60 mg in combination with vitamin C 160 mg manufactured by KAHIRA PHARM. and CHEM.IND.CO.CAIRO-EGYPT under the trade name of RUTA C 60^®^, three times daily for a period of 4 months in addition to their routine therapy.

The second group (group2) was constituted of 35 patients who received a dose of one capsule of vitamin C 500 mg manufactured by Hikma pharma S.A.E-Egypt under the trade name of C-Retard^®^, two times daily for a period of 4 months in addition to their routine therapy.

The dose of vitamin C in our study was selected based on the Ramos et al. study in which a dose of 1 g/day of vitamin C was supplemented to HD patients (Ramos and Martínez-Castelao, 2008).

Finally, the control group (group 3) was constituted of 35 patients who received their routine therapy.

The routine therapy was calcium-based phosphate binder (calcium acetate 700 mg = calcium 180 mg), calcimimetic agent (cinacalcet 30 mg), epoetin alpha injection, alfacalcidol (0.25 µg each other day), low dose aspirin (75 mg/QD), and antihypertensive medications: B-Blockers, Angiotensin converting enzyme inhibitor (captopril), calcium channel blockers (amlodipine), diuretics (hydrochlorothiazide), and methyldopa.

### 2.5 Clinical and laboratory data

Relevant information for all groups, such as the age, sex, weight, cause of end-stage renal disease, duration of end-stage renal disease and HD comorbidities were collected, routine biochemical data corresponding to collection date, such as hemoglobin, white blood cells, platelet counts, serum phosphorus, potassium, calcium, urea, and albumin were also obtained from routine medical records, body mass index (BMI), lipid profile including; total cholesterol (TC), triglycerides (TG), high-density lipoprotein cholesterol (HDL-C), and low-density lipoprotein cholesterol (LDL-C), and liver enzymes; alanine transaminase (ALT), and aspartate transaminase (AST) were determined.

In the current study, both oxidative stress and inflammation were determined through evaluation of levels of malondialdehyde (MDA), glutathione peroxidase (GPx), high sensitivity C-reactive protein (hs-CRP), tumor necrosis factor-alpha (TNF-α), and erythrocyte sedimentation rate (ESR), for all groups before and after intervention.

### 2.6 Patients’ follow-up

Follow-up of patients throughout the 16 weeks of the study was performed, where, patients were interviewed during their regular visits at the dialysis unit, which was three times weekly, and were educated regarding: medication side effects and medication adherence, the patients were asked to return the empty strips of the interventional medications to assess their adherence, and were asked about the incidence of any side and/or adverse effects to assess tolerability.

### 2.7 Laboratory measurements

Blood samples were withdrawn and collected in Vacutainer^®^ SST™ tubes (serum separator tubes) at baseline and at the end of the study from all groups just before the start of the hemodialysis session. Then, it was centrifuged at 3,000 rpm, for 10 min at 4^°^C, to separate sera and stored at -80°C until analysis for biochemical evaluation of MDA, GPx, hs-CRP, and TNF-alpha, lipid profile (TC, TG, HDL-c, and LDL-c), ALT, and AST. Besides, blood samples were also withdrawn at baseline and at the end of the study from all groups just before the start of the hemodialysis session for the measurement of erythrocyte sedimentation rate (ESR).

Study outcomes included the improvement of the following oxidative stress and inflammatory markers: MDA, GPx, hs-CRP, TNF-α, and ESR. The Tumor Necrosis Factor-alpha (TNF-α), MDA, and GPx were determined by enzyme-linked immunosorbent assay (ELISA) by using the human tumor necrosis factor-alpha ELISA Kit, Cat. No E0082Hu, MDA by using the human malondialdehyde ELISA KIT, Cat. No E1371Hu., and GPx by using the human glutathione peroxidase ELISA KIT, Cat. No. E3696Hu. All ELISA kits are manufactured by Bioassay Technology Laboratory BT LAB, China.

Concerning the high sensitivity C-reactive protein (hs-CRP), it was measured using an Elisa kit manufactured by Monocent, Inc. USA (Yudkin et al., 1999). All ELISA procedures were performed according to the manufacturer’s instructions, using a Thermo Scientific Multiskan FC Microplate Reader by Thermo Fisher Scientific (Skanlt Software 4.1).

Regarding the lipid profile, including: TC, LDL-C, HDL-C, and TG were determined using enzymatic-colorimetric tests manufactured by Human Gesellschaft fur Biochemica und Diagnostica mbH-Germany. All methods were performed according to the instructions of the manufacturer. The total cholesterol was assayed using an enzymatic-colorimetric method (CHOD-PAP-Method) (Schettler and Nussei, 1975). The high-density lipid (HDL) cholesterol was assayed using precipitant and standard, for use with the human cholesterol liquicolor Test Kit (Gordon et al., 1977). Triglyceride (TG) was measured using an enzymatic-colorimetric method (GPO-PAP-method) (Schettler and Nussei, 1975), while the concentration of the low-density lipid (LDL) cholesterol was calculated from the total cholesterol concentration and concentrations of both HDL-C and TG were determined according to Friedewald et al., (Friedewald et al., 1972).

Finally, Alanine transaminase (ALT) and aspartate transaminase (AST) were determined by using the International Federation of Clinical Chemistry (IFCC) kinetic methods (Bergmeyer et al., 1976; Bergmeyer, 1980), the liquiUV Test kits used for ALT and AST assays are manufactured by Human Gesellschaft fur Biochemica und Diagnostica mbH-Germany.

### 2.8 Ethics

The study was conducted in accordance with good clinical practice guidelines and the ethical principles in the Declaration of Helsinki (as revised in 2013). This clinical trial followed the CONSORT guidelines and ICMJE recommendations. The protocol was approved by the ethics committee of faculty of the pharmacy, Ain Shams University, Cairo, Egypt, which is registered at the Egyptain Ministry of Health (MOH) under registration number (26), moreover, the study has been registered on ClinicalTrials.gov: NCT04955145. The participants were informed about the study protocol and a written informed consent was obtained from all the patients prior to their participation in the study without any obligation to withdraw from the study if they wanted to.

### 2.9 Sample size calculation

The sample size was calculated by GPower v.3.1.9.4 (Erdfelder et al., 1996). According to Connelly (2008), the literature suggests that a pilot study sample should be 10% of the sample projected for the larger parent study (Connelly, 2008). Therefore, for a full-scale study, expecting a small effect size of 0.10, to reach a power of 80% to reject the null hypothesis, with three equally sized groups and an alpha of 0.05, the sample size for our pilot study would be expected to be 96.6 (approximated to 97) participants. To account for dropouts and equality of group sizes, we decided to recruit a total of 105 participants, equally divided among the three groups (35 participants in each group).

### 2.10 Statistical analysis

The statistical analysis was performed in R software (version 4.1.1). Two-sided *p*-values of less than 0.05 were considered to represent a statistically significant result. Shapiro-Wilk’s test was performed to test the normality of data across all treatment groups, and non-parametric tests were chosen given the violation of the normality assumption in at least one of the comparator groups in most instances. Electrolyte levels (Ca, K, and PO_4_), albumin levels, total leucocytic count, hemoglobin, platelet count, pre and post-dialysis urea, inflammatory and oxidative stress markers (MDA, GPx, hs-CRP, TNF-α, and ESR), cholesterol (LDL, HDL, TC, and TG), and hepatic transaminases were compared using the Kruskal–Wallis test before and after treatment across groups and were compared before and after treatment within each group using Wilcoxon’s signed-rank test.

## 3 Results

A total of 94 patients on maintenance HD completed the study and were analyzed. Eleven patients were lost to follow-up for the reasons described in Figure 1.

### 3.1 Baseline clinical data and demographics

At baseline, demographic and clinical characteristics were assessed, and there were no significant differences between the three study groups (Table 1) Statistical analysis of the baseline laboratory measurements presented in Table 2 showed a non-significant difference between the three studied groups, except for platelet count and hs C-reactive protein, with a *p*-value = 0.05 for both of them.

**TABLE 1:** Baseline demographic data and clinical characteristics of the studied groups.

	Control (*n* = 32)	RC (*n* = 31)	VC (*n* = 31)	Overall (*n* = 94)	*p* value
Demographics					
Age (years)					0.26^c^
Median (range)	44.0 (19.0–70.0)	55.0 (18.0–74.0)	50.0 (18.0–70.0)	52.0 (18.0–74.0)	
Gender					0.93^a^
Females	20 (62.5%)	19 (61.3%)	18 (58.1%)	57 (60.6%)	
Males	12 (37.5%)	12 (38.7%)	13 (41.9%)	37 (39.4%)	
BMI (kg/m^2^)					0.85^c^
Median (range)	25.2 (16.3–39.7)	27.7 (16.4–42.2)	24.7 (16.4–48.8)	26.1 (16.3–48.8)	
Comorbidities					
Thyroid disorder	4 (12.5%)	6 (19.4%)	10 (32.3%)	20 (21.3%)	0.15^b^
Hypertension	21 (65.6%)	20 (64.5%)	16 (51.6%)	57 (60.6%)	0.45^a^
Cardiovascular disease	4 (12.5%)	8 (25.8%)	5 (16.1%)	17 (18.1%)	0.40^b^
Arthritis	1 (3.1%)	2 (6.5%)	1 (3.2%)	4 (4.3%)	0.84^b^
Anemia	2 (6.2%)	1 (3.2%)	0 (0.0%)	3 (3.2%)	0.77^b^
Peptic ulcer disease	1 (3.1%)	1 (3.2%)	0 (0.0%)	2 (2.1%)	1.00^b^
Breast cancer	0 (0.0%)	1 (3.2%)	0 (0.0%)	1 (1.1%)	0.66^b^
Causes of ESRD					
Hypertension	19 (59.4%)	21 (67.7%)	18 (58.1%)	58 (61.7%)	0.70^a^
Vesicoureteral reflux	2 (6.2%)	1 (3.2%)	0 (0.0%)	3 (3.2%)	0.77^b^
Alport disease	1 (3.1%)	0 (0.0%)	0 (0.0%)	1 (1.1%)	1.00^b^
Autoimmune disease	2 (6.2%)	1 (3.2%)	0 (0.0%)	3 (3.2%)	0.77^b^
Diabetes mellitus	5 (15.6%)	6 (19.4%)	5 (16.1%)	16 (17.0%)	0.91^a^
Nephrolithiasis	1 (3.1%)	1 (3.2%)	1 (3.2%)	3 (3.2%)	1.00^b^
Analgesic abuse	4 (12.5%)	6 (19.4%)	6 (19.4%)	16 (17.0%)	0.74^b^
Idiopathic	2 (6.2%)	2 (6.5%)	2 (6.5%)	6 (6.4%)	1.00^b^
Congenital obstructive uropathy	0 (0.0%)	2 (6.5%)	3 (9.7%)	5 (5.3%)	0.20^b^
Renal fibrosis	1 (3.1%)	0 (0.0%)	0 (0.0%)	1 (1.1%)	1.00^b^
Gout treatment	1 (3.1%)	0 (0.0%)	0 (0.0%)	1 (1.1%)	1.00^b^
Nephritis	1 (3.1%)	3 (9.7%)	0 (0.0%)	4 (4.3%)	0.22^b^
Polycystic kidney disease	0 (0.0%)	1 (3.2%)	3 (9.7%)	4 (4.3%)	0.12^b^

a Pearson’s chi-squared test.

b Fisher’s exact test.

c Kruskal–Wallis test.

RC, RUTA C; VC, vitamin C; BMI, body mass index; ESRD, end-stage renal disease; n, number of patients.

**TABLE 2:** Baseline values of laboratory’ measurements of the studied groups.

	Control (*n* = 32)	RC (*n* = 31)	VC (*n* = 31)	Overall (*n* = 94)	p* value
K (mEq/L)	4.9 (2.7–7.6)	4.9 (3.3–6.6)	5.0 (3.5–6.7)	4.9 (2.7–7.6)	0.69
Ca (mg/dl)	8.7 (0.3–11.1)	8.9 (7.7–10.3)	8.9 (6.7–10.0)	8.8 (0.3–11.1)	0.75
PO4 (mg/dl)	3.9 (0.2–9.3)	4.1 (1.5–8.0)	4.1 (2.1–8.3)	4.1 (0.2–9.3)	0.84
Albumin (g/dl)	3.7 (0.8–4.6)	3.8 (3.1–4.6)	3.9 (3.1–4.7)	3.8 (0.8–4.7)	0.41
WBC (× 10^3^/ul)	6.2 (3.1–11.8)	6.3 (2.5–12.3)	6.5 (2.3–14.6)	6.3 (2.3–14.6)	0.83
Hb (g/dl)	10.1 (6.5–13.2)	9.9 (7.4–13.9)	9.9 (6.1–13.4)	9.9 (6.1–13.9)	0.97
PLT (× 10^3^/ul)	226.5 (164.0–599.0)	221.0 (81.0–886.0)	188.0 (22.0–390.0)	219.5 (22.0–886.0)	0.05
Pre-dialysis urea (mg/dl)	55.5 (18.0–80.0)	53.0 (35.0–71.0)	53.0 (33.0–94.0)	54.0 (18.0–94.0)	0.88
Post-dialysis urea (mg/dl)	14.0 (1.0–31.0)	17.0 (1.0–28.0)	16.0 (1.0–28.0)	16.0 (1.0–31.0)	1.00
MDA (nmol/ml)	8.0 (5.0–24.0)	12.0 (5.0–70.0)	10.0 (3.0–65.0)	10.0 (3.0–70.0)	0.23
GPx (ng/ml)	18.0 (6.0–36.0)	20.0 (6.0–55.0)	18.0 (8.0–48.0)	18.0 (6.0–55.0)	0.18
hs-CRP (mg/L)	7.1 (3.1–22.5)	5.5 (1.5–21.8)	6.3 (3.1–28.5)	6.3 (1.5–28.5)	0.05
TNF-α (ng/L)	180.0 (70.0–310.0)	210.0 (70.0–660.0)	190.0 (100.0–750.0)	190.0 (70.0–750.0)	0.21
ESR (mm/h)	22.5 (10.0–40.0)	20.0 (5.0–40.0)	22.0 (10.0–60.0)	20.0 (5.0–60.0)	0.25
TC (mg/dl)	204.0 (130.0–253.0)	200.0 (120.0–275.0)	200.0 (137.0–275.0)	200.5 (120.0–275.0)	0.93
LDL (mg/dl)	150.0 (66.0–196.0)	151.0 (85.0–203.0)	141.0 (100.0–203.0)	150.0 (66.0–203.0)	0.34
HDL (mg/dl)	34.0 (25.0–42.0)	35.0 (22.0–43.0)	33.0 (25.0–46.0)	35.0 (22.0–46.0)	0.85
TG (mg/dl)	124.0 (88.0–240.0)	134.0 (98.0–213.0)	134.0 (96.0–210.0)	132.5 (88.0–240.0)	0.27
ALT (U/l)	28.5 (13.0–49.0)	28.0 (13.0–62.0)	29.0 (10.0–52.0)	28.5 (10.0–62.0)	0.99
AST (U/l)	29.0 (13.0–45.0)	30.0 (18.0–43.0)	31.0 (15.0–44.0)	30.0 (13.0–45.0)	0.23

*Kruskal–Wallis test.

K, potassium; Ca, Calcium; PO4, phosphate; WBC, white blood cell; Hb, hemoglobin; PLT, platelet count; MDA, malondialdehyde; GPx, glutathione peroxidase; hs-CRP, high-sensitivity C-reactive protein; TNF-α, tumor necrosis factor-alpha; ESR, erythrocyte sedimentation rate; TC, total cholesterol; LDL, low-density lipoprotein cholesterol; HDL, high-density lipoprotein cholesterol; TG, triglycerides; ALT, alanine transaminase; AST, aspartate transaminase; RC, RUTA C; VC, vitamin C; n, number of patients.

### 3.2 End of the study assessment—Drug efficacy

#### 3.2.1 Antioxidant effect

The data represented in Table 4 showed that at the end of the study, evaluation of the antioxidant effect through measurement of GPx showed a significant increase in the vitamin C group (*p* = 0.001) compared to its baseline results. However, a non-significant difference was reported in the other groups when compared to the baseline values (Table 3). In addition, no significant differences were found among the three groups. Concerning the serum level of MDA, a significant decrease in all groups compared to the baseline values, with a non-significant difference among the three groups at the end of the study was reported (Table 4).

**TABLE 3:** Values of laboratory parameters and markers of oxidative stress before and at the end of the study period among the studied groups.

Lab test	Control (*n* = 32)			Rutin (*n* = 31)			Vitamin C (*n* = 31)		
	Baseline	Post	p* value	Baseline	Post	p* value	Baseline	Post	p* value
K (mEq/L)	4.9 (2.7–7.6)	5.25 (4.2–6.3)	0.267	4.9 (3.3–6.6)	5 (3.7–6.1)	0.770	5 (3.5–6.7)	5.3 (2.5–7.1)	0.170
Ca (mg/dl)	8.7 (0.3–11.1)	8.65 (7–10.2)	0.186	8.9 (7.7–10.3)	8.4 (7.7–10.7)	<0.001	8.9 (6.7–10)	8.6 (7–9.9)	0.214
PO4 (mg/dl)	3.9 (0.2–9.3)	4.5 (1.3–8.6)	0.245	4.1 (1.5–8)	4.4 (1.1–7.4)	0.781	4.1 (2.1–8.3)	4.3 (1.2–8.9)	0.883
WBC (× 10^3^/ul)	6.15 (3.1–11.8)	6.35 (3.6–10.9)	0.495	6.3 (2.5–12.3)	7.1 (2.2–14.1)	0.084	6.5 (2.3–14.59)	7.4 (1.7–13)	0.313
HB (g/dl)	10.05 (6.5–13.2)	10.5 (6.5–13)	0.226	9.9 (7.4–13.9)	9.7 (7.6–14)	0.860	9.9 (6.1–13.4)	10.1 (8.2–13.2)	0.230
PLT (× 10^3^/ul)	226.5 (164–599)	224.5 (121–625)	0.399	221 (81–886)	231 (50–718)	0.967	188 (22–390)	204 (92–476)	1.000
Pre-dialysis urea (mg/dl)	55.5 (18–80)	67 (20–111)	0.004	53 (35–71)	68 (38–97)	0.001	53 (33–94)	72 (20–109)	<0.001
Post-dialysis urea (mg/dl)	14 (1–31)	19 (1–35)	0.136	17 (1–28)	24 (2–47)	0.001	16 (1–28)	23 (4–50)	<0.001
MDA (nmol/ml)	8 (5–24)	5.75 (2.5–10)	<0.001	12 (5–70)	6 (2.5–11)	<0.001	10 (3–65)	5 (3–11)	<0.001
GPx (ng/ml)	18 (6–36)	22 (7–54)	0.127	20 (6–55)	24 (13–72)	0.389	18 (8–48)	24 (13–62)	0.001
CRP (mg/L)	7.1 (3.1–22.5)	7.2 (4–19.8)	0.374	5.5 (1.5–21.8)	7.5 (1–28.5)	0.098	6.3 (3.1–28.5)	7.5 (2.2–10)	0.433
TNF-a (ng/L)	180 (70–310)	40 (25–110)	<0.001	210 (70–660)	40 (25–110)	<0.001	190 (100–750)	40 (20–90)	<0.001
ESR (mm/h)	22.5 (10–40)	35 (20–80)	<0.001	20 (5–40)	25 (5–60)	0.001	22 (10–60)	30 (15–60)	0.003
TC (mg/dl)	204 (130–253)	171.5 (115–202)	<0.001	200 (120–275)	177 (113–205)	<0.001	200 (137–275)	167 (125–208)	<0.001
LDL (mg/dl)	150 (66–196)	108 (54–141)	<0.001	151 (85–203)	110 (49–136)	<0.001	141 (100–203)	105 (52–144)	<0.001
HDL (mg/dl)	34 (25–42)	37.5 (27–45)	0.017	35 (22–43)	38 (28–45)	0.019	33 (25–46)	36 (25–44)	0.365
TG (mg/dl)	124 (88–240)	132.5 (104–164)	0.984	134 (98–213)	125 (96–170)	0.232	134 (96–210)	130 (87–186)	0.118
Albumin (g/dl)	3.7 (0.8–4.6)	3.8 (2.5–4.6)	0.268	3.8 (3.1–4.6)	3.8 (3.2–4.7)	0.712	3.9 (3.1–4.7)	3.9 (3.4–4.6)	0.985
ALT (U/l)	28.5 (13–49)	22 (13–45)	0.234	28 (13–62)	22 (13–48)	0.173	29 (10–52)	23 (18–38)	0.362
AST (U/l)	29 (13–45)	29.5 (15–44)	0.610	30 (18–43)	28 (14–43)	0.152	31 (15–44)	29 (18–40)	0.144

*Wilcoxon signed rank-sum test.

K, potassium; Ca, calcium; PO4, phosphate; WBC, white blood cell; Hb, hemoglobin; PLT, platelet count; MDA, malondialdehyde; GPx, glutathione peroxidase; hs-CRP, high-sensitivity C-reactive protein; TNF-α, tumor necrosis factor-alpha; ESR, erythrocyte sedimentation rate; TC, total cholesterol; LDL, low-density lipoprotein cholesterol; HDL, high-density lipoprotein cholesterol; TG: triglycerides; ALT, alanine transaminase; AST, aspartate transaminase; RC: RUTA C; VC. vitamin C; n. number of patients.

**TABLE 4:** Values of laboratory parameters and markers of oxidative stress at the end of the study period among the studied groups.

	Control (*n* = 32)	RC (*n* = 31)	VC (*n* = 31)	Overall (*n* = 94)	*p* value
K (mEq/L)	5.2 (4.2–6.3)	5.0 (3.7–6.1)	5.3 (2.5–7.1)	5.2 (2.5–7.1)	0.43
Ca (mg/dl)	8.6 (7.0–10.2)	8.4 (7.7–10.7)	8.6 (7.0–9.9)	8.6 (7.0–10.7)	0.75
PO4 (mg/dl)	4.5 (1.3–8.6)	4.4 (1.1–7.4)	4.3 (1.2–8.9)	4.3 (1.1–8.9)	0.66
Albumin (g/dl)	3.8 (2.5–4.6)	3.8 (3.2–4.7)	3.9 (3.4–4.6)	3.8 (2.5–4.7)	0.25
WBC (× 103/ul)	6.3 (3.6–10.9)	7.1 (2.2–14.1)	7.4 (1.7–13.0)	7.1 (1.7–14.1)	0.72
HB (g/dl)	10.5 (6.5–13.0)	9.7 (7.6–14.0)	10.1 (8.2–13.2)	10.0 (6.5–14.0)	0.31
PLT (× 103/ul)	224.5 (121.0–625.0)	231.0 (50.0–718.0)	204.0 (92.0–476.0)	218.5 (50.0–718.0)	0.38
Pre-dialysis urea (mg/dl)	67.0 (20.0–111.0)	68.0 (38.0–97.0)	72.0 (20.0–109.0)	68.5 (20.0–111.0)	0.47
Post-dialysis urea (mg/dl)	19.0 (1.0–35.0)	24.0 (2.0–47.0)	23.0 (4.0–50.0)	22.0 (1.0–50.0)	0.05
MDA (nmol/ml)	5.8 (2.5–10.0)	6.0 (2.5–11.0)	5.0 (3.0–11.0)	5.0 (2.5–11.0)	0.54
GPx (ng/ml)	22.0 (7.0–54.0)	24.0 (13.0–72.0)	24.0 (13.0–62.0)	24.0 (7.0–72.0)	0.29
CRP (mg/L)	7.2 (4.0–19.8)	7.5 (1.0–28.5)	7.5 (2.2–10.0)	7.5 (1.0–28.5)	0.74
TNF-α (ng/L)	40.0 (25.0–110.0)	40.0 (25.0–110.0)	40.0 (20.0–90.0)	40.0 (20.0–110.0)	0.52
ESR (mm/h)	35.0 (20.0–80.0)	25.0 (5.0–60.0)	30.0 (15.0–60.0)	35.0 (5.0–80.0)	0.09
TC (mg/dl)	171.5 (115.0–202.0)	177.0 (113.0–205.0)	167.0 (125.0–208.0)	173.0 (113.0–208.0)	0.88
LDL (mg/dl)	108.0 (54.0–141.0)	110.0 (49.0–136.0)	105.0 (52.0–144.0)	107.5 (49.0–144.0)	0.74
HDL (mg/dl)	37.5 (27.0–45.0)	38.0 (28.0–45.0)	36.0 (25.0–44.0)	37.0 (25.0–45.0)	0.63
TG (mg/dl)	132.5 (104.0–164.0)	125.0 (96.0–170.0)	130.0 (87.0–186.0)	128.0 (87.0–186.0)	0.71
ALT (U/l)	22.0 (13.0–45.0)	22.0 (13.0–48.0)	23.0 (18.0–38.0)	22.0 (13.0–48.0)	0.99
AST (U/l)	29.5 (15.0–44.0)	28.0 (14.0–43.0)	29.0 (18.0–40.0)	29.0 (14.0–44.0)	0.79

*Kruskal–Wallis test.

K, potassium; Ca, calcium; PO4, phosphate; WBC, white blood cell; Hb, hemoglobin; PLT, platelet count; MDA, malondialdehyde; GPx, glutathione peroxidase; hs-CRP, high-sensitivity C-reactive protein; TNF-α, tumor necrosis factor-alpha; ESR, erythrocyte sedimentation rate; TC, total cholesterol; LDL, low-density lipoprotein cholesterol; HDL, high-density lipoprotein cholesterol; TG, triglycerides; ALT, alanine transaminase; AST, aspartate transaminase; RC, RUTA C; VC, vitamin C; n, number of patients.

#### 3.2.2 Anti-inflammatory effect

Concerning the evaluation of the anti-inflammatory effect of the interventional drugs, it was clear from the results presented in (Tables 3, 4) that the serum hs-CRP has increased in all groups with a non-significant difference, although there was a smaller increase in the vitamin C group, from 6.3 (3.1–28.5) mg/L to 7.5 (2.2–10) mg/L (*p* = 0.433) compared to a higher increase in the Ruta C group, from 5.5 (1.5–21.8) mg/L to 7.5 (1–28.5) mg/L (*p* = 0.098).

Moreover, the values of serum TNF-α had decreased significantly (*p* < 0.001) among the three groups in comparison to their baseline values (Table 3), on the other hand there was no significant difference shown on comparing the three groups with each other (*p* = 0.52).

Additionally, the results presented in (Table 4) showed a significant increase in the ESR values among the three groups compared to baseline values (Table 3),where, a higher increase in values was observed in the control group from 22.5 (10–40) mm/hr to 35 (20–80) mm/hr, *p* < 0.001 compared to the interventional groups, which showed a lower increase from 22 (10–60) mm/hr to 30 (15–60) mm/hr, *p* = 0.003, and from 20 (5–40) mm/hr to 25 (5–60) mm/hr, *p* = 0.001, in vitamin C and RUTA C, respectively. However, there was a non-significant difference among the three groups, as shown in (Table 4).

#### 3.2.3. Effect of vitamin C and rutin/vitamin C on patients’ lipid profile

Regarding the results of the lipid profile, it was apparent from (Tables 3, 4) that there was a non-significant difference among the three groups, with an increase in HDL-C levels in all of them, this increase was significant only in the RUTA C and control groups [from 35 (22–43) mg/dl to 38 (28–45) mg/dl, *p* = 0.019, and from 34 (25–42) mg/dl to 37.5 (27–45) mg/dl, *p* = 0.017, respectively]. On the other hand, there was a non-significant increase in the vitamin C group, [from 33 (25–46) mg/dl to 36 (25–44) mg/dl, *p* = 0.365].Besides, the statistical analysis result of the LDL-C levels showed a significant decrease from 141 (100–203) mg/dl to 105 (52–144) mg/dl, *p* < 0.001, from 151 (85–203) mg/dl to 110 (49–136) mg/dl, *p* < 0.001, and from 150 (66–196) mg/dl to 108 (54–141) mg/dl, *p* < 0.001 for the vitamin C, RUTA C, and control groups, respectively, before and at the end of the study, moreover, a non-significant decrease was obtained among the three studied groups.

Concerning the total cholesterol (TC) levels, there was a non-significant difference among the studied groups before and at the end of the study, while there was a significant decrease from 200 (137–275) mg/dl to 167 (125–208) mg/dl, *p* < 0.001, from 204 (130–253) mg/L to 171.5 (115–202) mg/dl, *p* < 0.001, and from 200 (120–275) mg/dl to 177 (113–205) mg/dl, *p* < 0.001 for vitamin C, control, and RUTA C groups, respectively.

Triglycerides results showed a non-significant difference among the three groups a non-significant decrease in both the vitamin C and RUTA C groups, and a non-significant increase in the control group were observed before and at the end of the study.

#### 3.2.4 Effect of vitamin C and rutin/vitamin C on laboratory parameters

Statistical analysis of the obtained results showed a non-significant difference among the studied groups with regard to the values of, ALT, AST, Albumin, Hb, K, Ca, PO4, WBC, and PLT count at the end of the study Tables 3, 4.

#### 3.2.5 Assessment of safety and tolerability

Regarding safety and tolerability of the interventional drugs, it was reported that one patient suffered from gastrointestinal tract (GIT) upset and one patient recorded a decrease in blood pressure on administration of RUTA C. While with the vitamin C supplement alone, only one patient suffered from GIT upset and vomiting from the beginning of its administration.

## 4 Discussion

Malondialdehyde (MDA) is the end product of lipid peroxidation and is used as a great oxidative stress marker (Chen et al., 2011), MDA-LDL/LDL-C ratio has been shown to have a significant elevation in HD patients and could be considered as a potential risk factor for cardiovascular events in those patients (Asamiya et al., 2013). Also, a decrease in the levels of GSH or GPx has been seen in CKD patients (Duni et al., 2017). Vera et al. (2018) suggested that the potentiation of the GPx pathway could be future promising method to reduce the endothelial dysfunction induced in CKD patients, as GPx is known to minimize lipid peroxidation of the cell membranes accompanying OS.

In the current study, the administration of oral vitamin C alone (group 2) showed a significant increase in the serum levels of glutathione peroxidase GPx from 18 (8–48) ng/ml at baseline to 24 (13–62) ng/ml (*p* = 0.001) at the end of the 16-week study period compared to a non-significant difference in both groups 1 and 3. To the best of our knowledge, this observation was the first one to be documented compared to the other previously performed studies, which lacked such a newly detected elevation in HD patients supplemented with vitamin C.

Wen et al., demonstrated that glutathione (GSH) values were increased by 28% after 4 weeks of supplementation with 1000 mg/day of vitamin C compared to the control (*p* > 0.001) (Wen et al., 1997), which was in accordance with our results.

On the other hand, Fumeron et al. (2005) reported a non-significant change in GSH levels upon the administration of 250 mg of vitamin C for 8 weeks compared to controls.

In addition, Martins et al. (2021) reported a non-significant decrease in GPx levels when patients were given 0.250 g of vitamin C three times/week for 8 weeks. The discrepancy between the previously mentioned studies’ results and the current study ones might be due to their shorter length of study intervention (8 weeks) compared to ours (16 weeks), about double the study period.

In the present study, after 16 weeks of supplementation, the data showed a significant reduction in MDA levels at the end of the study in the three studied groups compared to their baseline levels (*p* > 0.001). Interestingly, the percentage change in MDA levels was reduced by 50% in interventional groups and by only 28% in the control group. On considering the vitamin C effect, our findings were consistent with those obtained by Sarandol et al. (2022), who reported that the supplementation of vitamin C showed a significant decrease in MDA.

Also, Abdollahzad et al. (2009) study results showed a decrease in the level of serum MDA in the vitamin C group after 12 weeks of supplementation of 0.25 g three times/week and a significant difference in mean MDA changes between the vitamin C group and placebo group. In addition, Candan et al. (2002) reported that MDA levels significantly decreased in vitamin C supplemented patients when compared with those receiving placebo at the end of 3 months.

In the present study, there was a non-significant difference in hs-CRP levels among the three groups at the end of the study. Interestingly, when we compared the interquartile range values at baselines 6.3 (3.1–28.5) with that at end of the study 7.5 (2.2–10) of the vitamin C group, we found 75% of patients had hs-CRP levels ≤10 mg/L after treatment compared to ≤28.5 mg/L before treatment, this result may reflects the tendency of vitamin C to decrease hs-CRP levels in the majority of patients, and it may be due to the differences in their duration of dialysis, wide range of the age and comorbidities of the patients that the rest of patients resembling 25% did not show such a large reduction in their hs-CRP’ levels.

In agreement with our results, Fumeron et al. (2005) reported no effect on plasma hs-CRP after oral supplementation of vitamin C every second day for a period of 2 months, although the level of vitamin C was increased. However, in contrast with Biniaz et al. (2014) who demonstrated a significant fall in median CRP level in a group of patients on hemodialysis after 2 months of supplementation with vitamin C 250 mg three times/week.

In the current study, although the three studied groups did not show any significant difference regarding TNF-α levels at the end of our study, all groups showed a significant decrease in the TNF-α levels compared to their baseline values.

When we compared median TNF-α values of the three groups at end of the study with their baseline values, we found that administration of rutin/vitamin C combination (Ruta C) causes a reduction of TNF-α levels in 50% of the patients from 210 ng/L to 40 ng/L indicating the ability of rutin to alleviate the inflammatory response and manifested itself to produce a small synergistic effect with vitamin C, when given together as a combination, and also as a promise anti-inflammatory treatment attenuating inflammation and oxidative stress in HD patients.

On the other side, we could not ignore the effect of vitamin C alone (vitamin C group) in decreasing the TNF-α level in 25% of patients to ≤20 at the end of the study compared to ≤100 at baseline in accordance with those reported by Khosroshahi et al. (2011) that TNF-α levels reduced significantly in HD patients after intravenous administration of Vitamin C with 500 mg two times/week after each dialysis session for 8 weeks.

In fact, there were limited data in the literature which focused on the anti-inflammatory effect of vitamin C in HD patients. The anti-inflammatory mechanism of vitamin C was clarified by its inhibitory effect on Nuclear Factor Kappa B (NF-kB) which is responsible for the activation of a number of pro-inflammatory cytokines such as TNF-α (Carr and Maggini, 2017).

Concerning the non-significant increase in ESR values in the three studied groups which were observed at the end of our study period, with a higher increase in values observed in the control group compared to the interventional groups revealed that although vitamin C failed to decrease ESR significantly, it succeeded in avoiding its increase above the highest baseline value (60 mm/h). Moreover, it was suggested that the longer duration of patients’ intake of vitamin C might have induced favorable actions supported by those reported by Khosroshahi et al. (2011) for the reduction in ESR level in their study group supplemented with vitamin C.

Considering the effect of vitamin C on lipid profile levels, our results were in accordance with a meta-analysis of 13 randomized controlled trials, which concluded that the administration of vitamin C at least 0.5 g/d for a minimum of 4 weeks can result in a significant decrease in serum LDL-C and reported a non-significant elevation of serum HDL-C with a significant fall in serum triglycerides (McRae, 2008). In addition, our results of the vitamin C group were supported by those found by El Mashad et al. (2016) where, a significant decrease in the levels of cholesterol and LDL after 12 weeks of supplementation with vitamin C failed to agree with their TG results.

## 5 Conclusion

From the results obtained in this study, it can be concluded that oral administration of 1 g/day of vitamin C for a period of 16 weeks resulted in the reduction of serum levels of malondialdehyde (MDA) and elevation of glutathione peroxidase (GPx) levels among hemodialysis patients (HD). Moreover, rutin had a favorable synergistic effect with vitamin C in reducing TG and TNF-α levels, and increasing HDL-C level. Rutin might provide a positive future result associated with promise as an anti-inflammatory supplementation, attenuating inflammation and oxidative stress in HD patients when combined with vitamin C.

## 6 Recommendations

A multi-center randomized controlled study with a relatively longer duration is required to confirm the role of vitamin C in improving the inflammatory status of HD patients. Moreover, a long-term trials and larger doses as well are recommended to explore the impact of rutin supplementation on the oxidative stress and inflammatory markers in ESRD population. Finally, future studies are to include detection of cytokine levels as well as broader investigations of the anti-inflammatory effect of vitamin C and rutin in HD patients.

## Data Availability

The raw data supporting the conclusion of this article will be made available by the authors without undue reservation.
